# Intratumor heterogeneity of PD-L1 expression in head and neck squamous cell carcinoma

**DOI:** 10.1038/s41416-019-0449-y

**Published:** 2019-04-10

**Authors:** Jacob H. Rasmussen, Giedrius Lelkaitis, Katrin Håkansson, Ivan R. Vogelius, Helle H. Johannesen, Barbara M. Fischer, Søren M. Bentzen, Lena Specht, Claus A. Kristensen, Christian von Buchwald, Irene Wessel, Jeppe Friborg

**Affiliations:** 1 Department of Otorhinolaryngology, Head and Neck Surgery and Audiology, Rigshospitalet, University of Copenhagen, Copenhagen, Denmark; 2 Department of Pathology, Rigshospitalet, University of Copenhagen, Copenhagen, Denmark; 3 Department of Oncology, Section of Radiotherapy, Rigshospitalet, University of Copenhagen, Copenhagen, Denmark; 4 Department of Clinical Physiology, Nuclear Medicine & PET, PET & Cyclotron Unit, Rigshospitalet University of Copenhagen, Copenhagen, Denmark; 5 PET Centre, School of Biomedical Engineering and Imaging Science, Kings College London, Guy’s & St. Thomas Hospital, London, UK; 6 Division of Biostatistics and Bioinformatics, University of Maryland Greenebaum Cancer Center, and Department of Epidemiology and Public Health, University of Maryland School of Medicine, Baltimore, USA

**Keywords:** Tumour heterogeneity, Predictive markers

## Abstract

Intratumor heterogeneity may contribute to the ambiguous clinical results on PD-L1 status as a predictor for immunotherapy response in patients with HNSCC. This decreases the utility of PD-L1 expression from single tumour biopsies as a predictive biomarker. In this prospective study, intratumor heterogeneity of PD-L1 expression in HNSCC was investigated with both Tumour Proportion Score (TPS) and Combined Positive Score (CPS). Thirty-three whole surgical specimens from 28 patients with HNSCC were included. PD-L1 expression in six random core biopsies from each surgical specimen was used to assess the concordance between multiple biopsies and the negative predictive value of a single negative core biopsy. With 1% cut off, 36% of the specimens were concordant with TPS and 52% with CPS. With a 50% cut-off value the concordance was 70% with TPS and 55% with CPS. Defining a tumour as positive if just a single-one of the biopsies was positive, the negative predictive value (NPV) of a single negative core biopsy was 38.9 and 0% (1% cut off), and 79.9% and 62.8% (50% cut off) for TPS and CPS, respectively. In conclusion, PD-L1 positivity varies markedly within the tumour, both with TPS and CPS, challenging the utility of this biomarker.

## Background

Head and neck squamous cell carcinoma (HNSCC) is a biologically and clinically heterogenous disease.^1^ Primary treatment includes surgery and/or radiotherapy, with or without concomitant chemotherapy, but ~15–50% of the patients experience treatment failure,^2^ HPV association being the most important prognostic factor. Patients with recurrent or metastatic disease have a very poor prognosis and systemic treatment with the combination of cetuximab, platinum and fluorouracil is standard first line treatment.^3^ Recently, immune checkpoint inhibitors, i.e. antibodies targeting the programmed cell death protein-1 (PD-1)/programmed death ligand 1 (PD-L1) signalling pathway have been approved by the FDA for second-line treatment of HNSCC.^4^ Nivolumab, a PD-1 inhibitor, demonstrated improved response rate and overall survival compared to conventional chemotherapy^5,6^ in a randomised phase III trial. Pembrolizumab, another PD-1 inhibitor, yielded response rates comparable to nivolumab in phase I, II and III studies.^7,8^ These treatments are costly, have side effects,^9,10^ and not all patients respond to treatment. This emphasises the need for predictive biomarkers and there is evidence suggesting a better response to treatment in patients whose tumours express PD-L1.^6,11^ However, clinical response has also occurred in patients with PD-L1 negative tumours and, conversely, PD-L1 expressing tumours have not responded to treatment. FDA has approved nivolumab and pembrolizumab for use in head and neck cancer patients with disease progression on or after platinum-based therapy, and nivolumab has been approved by EMA for use in Europe under a similar indication. Pembrolizumab, however, is approved only for adults whose tumours express PD-L1 with a ≥50% Tumour Proportion Score (TPS) and progressing on or after platinum-containing chemotherapy. Also, a Combined Positive Score (CPS) for PD-L1 expression has been shown to be predictive of benefit in recent studies in HNSCC.^7^ In other tumour types (e.g. non-small cell lung cancer and urothelial carcinoma), immunotherapy is approved only for patients with PD-L1 positive tumours. However, the cut-off value for PD-L1 expression is controversial, as demonstrated in a recent study in non-small cell lung cancer.^12^ Intratumor heterogeneity may be part of the explanation for this controversy and could invalidate the use of PD-L1 expression as a predictive marker for treatment selection. Thus, intratumor heterogeneity may have a direct impact on patient treatment not only for patients with HNSCC.

There are at least three main reasons for variability in PD-L1 expression between studies, between patients, and within tumours. First, technical reasons: different protocols for immunohistochemical staining use different antibodies with varying binding affinities, different scoring systems and criteria for positivity.^13,14^ Second, clinical reasons: scoring will depend on biopsy quality and inherently be subject to interobserver variability.^15,16^ Thirdly, biological reasons: PD-L1 expression varies within a given tumour.^17,18^ Expression of PD-L1 is usually assessed from single tumour biopsies, which will be affected by intratumor heterogeneity. To our knowledge, no study has investigated the intratumor heterogeneity in PD-L1 expression in head and neck cancer.

## Methods

### Patients and specimens

In this prospective study, patients with HNSCC referred for curative surgery were enroled. Only patients with tumour diameters >1.5 cm were included. No patient received immunotherapy. The study was approved by the Regional Committee on Health Research Ethics, approval number H-16049387 and the study was performed in accordance with the Declaration of Helsinki.

### Histology and immunohistochemistry

**Fig. 1:**
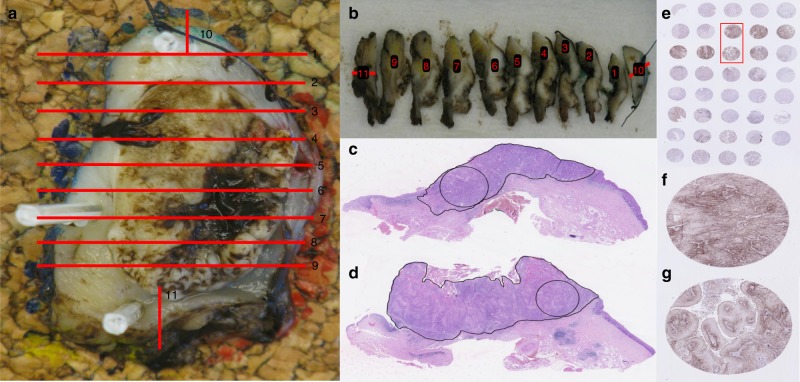
**a**–**g** illustrates the workflow and histologic processing in the study. a shows how the lesions were sectioned contiguously into tumour blocks, yielding 11 tumour blocks in this particularly case. Each red line on (**a**) corresponds to the specific tumour block number in Fig. 2b. Six blocks were selected randomly for further histologic processing. **c** and **d** depicts the 4-µm section stained with haematoxylin and eosin from block 2 and block 8. The black circle indicates from where the 3 mm core biopsy was performed. **e** illustrates a sectioned from a tissue micro array block stained for PD-L1 expression. **f** and **g** are the two cores marked with the red square in 1E and correspond the two cores in shown in **c**, **d**

Figure 1 illustrates the workflow in the histologic processing. All tumours were removed en bloc and after surgery all specimens were formalin fixated, embedded in paraffin and sectioned in 2–3 mm consecutive tissue blocks (Fig. 1a, b). Six tissue blocks from each specimen were selected randomly and core biopsied in an area with representative tumour tissue assessed from a 4-µm section stained with haematoxylin and eosin (H&E) (Fig. 1c, d). A 3 mm wide core biopsy was taken from each of the selected tumour blocks and used to construct tissue micro array (TMA) blocks (Fig. 1e). 4-µm sections were made from the TMA blocks and stained for H&E and p40 (platform Dako Omnis, clone BC28, code ACI3066C, mouse monoclonal anti-human, 1 + 50, Biocare Medical, Pacheco CA, USA) to assess biopsy quality as percentage tumour in the core biopsy and percentage of vital squamous tumour cells in the biopsy. PD-L1 expression was assessed as TPS and as CPS using platform Autostainer Link 48, clone 28-8, pharmDx kit, rabbit monoclonal anti-human, 1 + 200, Dako (Fig. 1f–g). Discordance or concordance in dichotomised PD-L1 positivity was estimated using 1% and 50% as cut-off values with both TPS and CPS.

## Results

### Patients and specimens

Overall twenty eight patients were included, sixteen males and twelve females. Sixteen patients had a tumour in the oral cavity, four in the oropharynx (one p16 positive), three in the hypopharynx, two in the maxillary sinus, and three had only tumours in a lymph node. Five patients had both a primary lesion and a lymph node metastasis yielding a total of 33 lesions. Two of the specimens only had four tumour blocks and another two only had five tumour blocks yielding 192 tumour blocks for TMA construction.

### PD-L1 expression

**Fig. 2:**
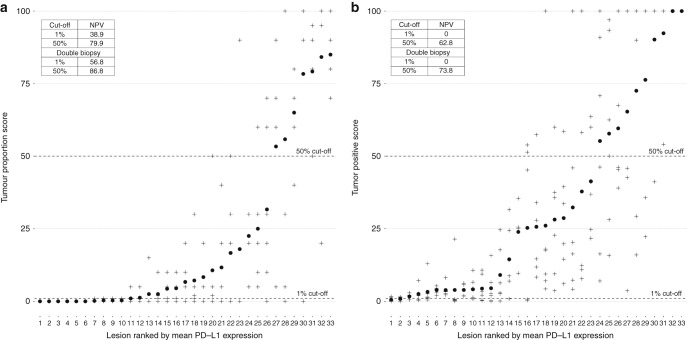
**a** shows a scatter plot of the PD-L1 score in each biopsy with tumour proportion score (TPS) and **b** shows the PD-L1 expression with combined positive score (CPS). In **a** and **b** the *y*-axis depicts the PD-L1 score from 0–100%. The *x*-axis depicts the 33 lesions ranked by mean PD-L1 score marked with a black circle for TPS and CPS, respectively. The crosses depict the actual score from each of the six biopsies. As an example, in lesion 1 and 2 all biopsies scored 0% in **a**. The black dashed lines marks 1% cut off and 50% cut-off values. The insert in the upper left corner of both (**a**) and (**b**) depicts the negative predictive value of a single negative core biopsy using a 1% cut off and 50% cut off for positivity with TPS (2A) and CPS (2B) in case of one biopsy and with double biopsy. The ground truth is assumed to be that a tumour is positive if any of the six core biopsies are positive. For the five patients with two lesions, lesion 30, 15, 25, 7 and 2 is the primary lesions and lesion 32, 12, 26, 8 and 1 is the corresponding lymph node lesions in **a**. Lesion 29, 17, 23, 2 and 12 is the primary lesions in **b** and lesion 32, 15, 24, 5 and 13 the corresponding lymph node lesions

Figure 2a, b depicts the full PD-L1 score from each core biopsy with TPS and CPS, respectively. Using a 1% cut-off value to define positivity, 36% of the specimens were concordant (all positive or all negative) in the six biopsies from each lesion with TPS and 52% were concordant with CPS. With a 50% cut off, the concordance was higher at 70% with TPS and 54% with CPS. For each score and each threshold of positivity, we define the ground truth as the tumour being PD-L1 positive if any of the cores from the tumour specimen is positive. With this definition, the positive predictive value of a single biopsy is identical 100% regardless of whether TPS or CPS is used to score PD-L1 expression. The negative predictive value (NPV) of a single negative biopsy is 38.9% with TPS. The NPV of a single negative biopsy with CPS using 1% cut-off value was 0% (Fig. 2) as none of the 33 lesions studied here were negative for PD-L1 expression in all six core biopsies with this definition. With 50% cut-off value, the NPV = 79.9% with TPS and 62.8% with CPS (Fig. 2). The scatter plots in Fig. 2a, b illustrates that any clinical/biological cut-off value for positivity would still be subject to intratumor heterogeneity if assessed from a single biopsy. Performing double biopsies could increase the predictive value; the NPV with double biopsies was 56.8% and 86.8% with TPS and 0% and 73.8% for CPS with 1% and 50% cut-offs, respectively (Fig. 2), again calling the tumour negative if and only if both biopsies were negative on the assay. There was no significant correlation between concordance in PD-L1 expression and tumour volume on magnetic resonance imaging. Heterogeneity in PD-L1 expression was observed both in small and in large tumours (data not shown). In the five patients with both a primary lesion and a lymph node lesion, there were no significant differences in PD-L1 expression between the primary lesions and the lymph nodes. The actual values for the respective lesions are shown in Fig. 2a, b.

## Discussion

In this study, all biopsies were performed as 3 mm core biopsies from whole specimen tumour blocs (Fig. 1). The core biopsies were selected from areas with representative tumour tissue as verified by the pathologist. Clinical biopsies used to assess PD-L1 expression for first- or second-line treatment may very well contain less tumour tissue, which will increase the uncertainty further. One desired property for a predictive biomarker is a high NPV and although the NPV using a 50% cut off for positivity with TPS was better at 79.9%, the majority of lesions (64%) was classified as negative using this definition (Fig. 2a). Although CPS in some studies has shown to be predictive for response in patients with recurrent HNSCC, no lesions were true negative with a cut-off value at 1%. This study illustrates the challenges of using PD-L1 as a predictive biomarker. While reporting the actual TPS or CPS value may convey biological information, using this biomarker for clinical decision making still requires a binary classification into positive or negative lesions.

In conclusion, both with TPS and CPS the assessed PD-L1 positivity varies markedly within the tumour in this patient series (Fig. 2), which limits the utility of this biomarker. However, identifying the optimal cut point for discrimination between responders and non-responders to a specific agent is a clinical/biological problem.

Intratumor heterogeneity most likely contributes to the ambiguous results on PD-L1 status seen in the Checkmate^6^ and Keynote^8^ studies of HNSCC patients and challenges the use of PD-L1 expression from single tumour biopsies as a predictor for immunotherapy in HNSCC patients. In future trials, the use of repeated biopsies or multiple tumour sampling from head and neck tumours as well as reporting the actual value of PD-L1 expression could be considered for better prediction of tumour response to immune checkpoint inhibitors targeting the PD-1/PD-L1 signalling pathway.

## Data Availability

The dataset used and analysed during the current study is available from the corresponding author on reasonable request.

## References

[1] Network TCGA. (2015). Comprehensive genomic characterization of head and neck squamous cell carcinomas. Nature.

[2] Pignon JP, le Maître A, Maillard E, Bourhis J (2009). Meta-analysis of chemotherapy in head and neck cancer (MACH-NC): an update on 93 randomised trials and 17,346 patients. Radiother Oncol..

[3] Vermorken JB, Mesia R, Rivera F, Remenar E, Kawecki A, Rottey S (2008). Platinum-based chemotherapy plus cetuximab in head and neck cancer. N. Engl. J. Med..

[4] Szturz P, Vermorken JB (2017). Immunotherapy in head and neck cancer: aiming at EXTREME precision. BMC Med..

[5] Ferris RL, Blumenschein G, Fayette J, Guigay J, Colevas AD, Licitra L (2018). Nivolumab vs investigator’s choice in recurrent or metastatic squamous cell carcinoma of the head and neck: 2-year long-term survival update of CheckMate 141 with analyses by tumor PD-L1 expression. Oral Oncol..

[6] Ferris RL, Blumenschein G, Fayette J, Guigay J, Colevas AD, Licitra L (2016). Nivolumab for recurrent squamous-cell carcinoma of the head and neck. N. Engl. J. Med..

[7] Bauml J, Seiwert TY, Pfister DG, Worden F, Liu SV, Gilbert J (2017). Pembrolizumab for platinum- and cetuximab-refractory head and neck cancer: results from a single-arm, phase II study. J Clin Oncol.

[8] Cohen E. E., Harrington K. J., Le Tourneau C., Dinis J., Licitra L., Ahn M.-J. et al. LBA45_PRPembrolizumab (pembro) vs standard of care (SOC) for recurrent or metastatic head and neck squamous cell carcinoma (R/M HNSCC): Phase 3 KEYNOTE-040 trial. *Ann. Oncol*. 2017; **28**. 10.1093/annonc/mdx440.040.

[9] Harrington Kevin J, Ferris Robert L, Blumenschein George, Colevas A Dimitrios, Fayette Jérôme, Licitra Lisa, Kasper Stefan, Even Caroline, Vokes Everett E, Worden Francis, Saba Nabil F, Kiyota Naomi, Haddad Robert, Tahara Makoto, Grünwald Viktor, Shaw James W, Monga Manish, Lynch Mark, Taylor Fiona, DeRosa Michael, Morrissey Laura, Cocks Kim, Gillison Maura L, Guigay Joël (2017). Nivolumab versus standard, single-agent therapy of investigator's choice in recurrent or metastatic squamous cell carcinoma of the head and neck (CheckMate 141): health-related quality-of-life results from a randomised, phase 3 trial. The Lancet Oncology.

[10] Mehra R, Seiwert TY, Gupta S, Weiss J, Gluck I, Eder JP (2018). Efficacy and safety of pembrolizumab in recurrent/metastatic head and neck squamous cell carcinoma: pooled analyses after long-term follow-up in KEYNOTE-012. Br. J. Cancer.

[11] Chow LQM, Haddad R, Gupta S, Mahipal A, Mehra R, Tahara M (2016). Antitumor activity of pembrolizumab in biomarker-unselected patients with recurrent and/or metastatic head and neck squamous cell carcinoma: results from the phase Ib KEYNOTE-012 expansion cohort. J Clin Oncol.

[12] Antonia Scott J., Villegas Augusto, Daniel Davey, Vicente David, Murakami Shuji, Hui Rina, Kurata Takayasu, Chiappori Alberto, Lee Ki H., de Wit Maike, Cho Byoung C., Bourhaba Maryam, Quantin Xavier, Tokito Takaaki, Mekhail Tarek, Planchard David, Kim Young-Chul, Karapetis Christos S., Hiret Sandrine, Ostoros Gyula, Kubota Kaoru, Gray Jhanelle E., Paz-Ares Luis, de Castro Carpeño Javier, Faivre-Finn Corinne, Reck Martin, Vansteenkiste Johan, Spigel David R., Wadsworth Catherine, Melillo Giovanni, Taboada Maria, Dennis Phillip A., Özgüroğlu Mustafa (2018). Overall Survival with Durvalumab after Chemoradiotherapy in Stage III NSCLC. New England Journal of Medicine.

[13] Hirsch FR, McElhinny A, Stanforth D, Ranger-Moore J, Jansson M, Kulangara K (2017). PD-L1 immunohistochemistry assays for lung cancer: results from phase 1 of the blueprint PD-L1 IHC assay comparison project. J Thorac Oncol.

[14] Rimm DL, Han G, Taube JM, Yi ES, Bridge JA, Flieder DB (2017). A prospective, multi-institutional, pathologist-based assessment of 4 immunohistochemistry assays for PD-L1 expression in non–small cell lung cancer. JAMA Oncol.

[15] Wang Chiyun, Hahn Elan, Slodkowska Elzbieta, Eskander Antoine, Enepekides Danny, Higgins Kevin, Vesprini Danny, Liu Stanley K., Downes Michelle R., Xu Bin (2018). Reproducibility of PD-L1 immunohistochemistry interpretation across various types of genitourinary and head/neck carcinomas, antibody clones, and tissue types. Human Pathology.

[16] Rehman JA, Han G, Carvajal-Hausdorf DE, Wasserman BE, Pelekanou V, Mani NL (2017). Quantitative and pathologist-read comparison of the heterogeneity of programmed death-ligand 1 (PD-L1) expression in non-small cell lung cancer. Mod Pathol.

[17] McLaughlin J, Han G, Schalper KA, Carvajal-Hausdorf D, Pelekanou V, Rehman J (2016). Quantitative assessment of the heterogeneity of PD-L1 expression in non-small-cell lung cancer. JAMA Oncol.

[18] Bhaijee F, Anders RA (2016). PD-L1 expression as a predictive biomarker. JAMA Oncol.

